# Single loss of a *Trp53* allele triggers an increased oxidative, DNA damage and cytokine inflammatory responses through deregulation of IκBα expression

**DOI:** 10.1038/s41419-021-03638-3

**Published:** 2021-04-06

**Authors:** Laura Marruecos, Joan Manils, Cristina Moreta, Diana Gómez, Ingrid Filgaira, Anna Serafin, Xavier Cañas, Lluís Espinosa, Concepció Soler

**Affiliations:** 1 Cancer Research Program, CIBERONC Institut Hospital del Mar d’Investigacions Mèdiques (IMIM), Barcelona, Spain; 2 Unitat d’Immunologia, Departament de Patologia i Terapèutica Experimental, Facultat de Medicina i Ciències de la Salut, Institut de Neurociències, Universitat de Barcelona, L’Hospitalet de Llobregat, Barcelona, Spain; 3 Department of Immunology & Inflammation, Imperial College London, London, United Kingdom; 4 PCB Animal Facility, Parc Científic de Barcelona, Barcelona, Spain; 5 Vall d’Hebron Institute of Research, Barcelona, Spain; 6 Neuropharmacology & Pain Group, Neuroscience Program, Institut d’Investigació Biomèdica de Bellvitge - IDIBELL, L’Hospitalet de Llobregat, Spain

**Keywords:** Tumour-suppressor proteins, Stress signalling, Chronic inflammation

## Abstract

Dose of *Trp53*, the main keeper of genome stability, influences tumorigenesis; however, the causes underlying and driving tumorigenesis over time by the loss of a single p53 allele are still poorly characterized. Here, we found that single p53 allele loss specifically impacted the oxidative, DNA damage and inflammatory status of hematopoietic lineages. In particular, single *Trp53* allele loss in mice triggered oxidative stress in peripheral blood granulocytes and spleenocytes, whereas lack of two *Trp53* alleles produced enhanced oxidative stress in thymus cells, resulting in a higher incidence of lymphomas in the *Trp53* knockout (KO) mice compared with hemizygous (HEM). In addition, single or complete loss of *Trp53* alleles, as well as p53 downregulation, led to a differential increase in basal, LPS- and UVB-induced expression of a plethora of pro-inflammatory cytokine, such as interleukin-12 (*Il-12a*), TNFα (*Tnfa*) and interleukin (*Il-23a*) in bone marrow-derived macrophage cells (BMDMs) compared to WT cells. Interestingly, p53-dependent increased inflammatory gene expression correlated with deregulated expression of the NF-κB pathway inhibitor IκBα. Chromatin immunoprecipitation data revealed decreased p65 binding to *Nfkbia* in the absence of p53 and p53 binding to *Nfkbia* promoter, uncovering a novel crosstalk mechanism between p53 and NF-κB transcription factors. Overall, our data suggest that single *Trp53* allele loss can drive a sustained inflammatory, DNA damage and oxidative stress response that, over time, facilitate and support carcinogenesis.

## Introduction

The tumor suppressor *TP53* (Tumor Protein P53) is among the most frequently mutated genes in a plethora of tumors, with more than 25,000 mutations registered in the International Agency for Research on Cancer. This guardian of the genome plays an essential role in the response to DNA damage and cellular stress induced by external and internal insults, which in turn trigger p53 expression and activation^1,2^.

According to its functional relevance, p53 levels are regulated by multiple transcriptional, translational, and post-translational mechanisms. In non-stressed cells, p53 levels are kept relatively low by means of MDM2 activity (M*ouse Double Minute 2*), which ubiquitinates p53 leading to its proteasomal degradation. In response to cellular stressors (DNA damage, hypoxia or oxidative metabolism, among others), interaction between p53 and MDM2 is disrupted thus resulting in a quick increase in p53 stabilization and activity associated with additional post-transcriptional modifications such as sumoylation, phosphorylation, acetylation and glycosylation^1,2^. In this manner, p53 accumulates and translocates to the nucleus, where it regulates the expression of genes that control numerous cellular functions, such as cell cycle, apoptosis, differentiation, senescence, DNA repair, and oxidative stress^3–5^. In fact, p53 tumor suppressor activity mainly relies in its function as upstream regulator of inflammatory gene transcription and oncogene-induced cell death^6–8^. Consequently, p53 knockout (KO) mice (*Trp53*^*−/−*^) show a higher expression of pro-inflammatory cytokines than wild-type (WT) mice^9–11^, and are prone to chronic inflammation^12^ as well autoimmune diseases^13,14^. Moreover, about 25% of p53 KO mice died owing to unresolved spontaneous inflammatory responses^15^.

Supporting the importance of p53 levels regulation, single loss or inactivating mutations of a *Trp53* allele are sufficient to induce tumorigenesis, as it is observed in patients of Li-Fraumeni syndrome that carry a germinal mutation of p53 gene in about 70% of cases^16^. Importantly, the frequency and spectrum of tumors arising in *Trp53* hemizygous (HEM) and homozygous KO mice are radically different, as well as their life expectancy^15,17–24^. The mechanisms underlying these differences remain largely undefined. In this sense, whereas tumor development in HEM mice and Li-Fraumeni syndrome patients, has been associated with a loss of hemizygosity or to a dominant-negative effect of mutated p53^23,25–30^, around 50% of tumors in HEM mice retain a copy of the WT allele^20,23,25^, suggesting that reduced dosage of p53 is sufficient for cancer development.

To gain insight into the mechanisms behind *Trp53* dosage loss influence in tumor spectrum, we have analyzed the early and late tumor phenotypes arising in p53 HEM and KO mice. Moreover, we have determined the amount of oxidative stress, DNA damage and inflammation, which are relevant hallmarks on the onset and evolution of carcinogenesis, under conditions of single and complete *Trp53* allele loss. Our results show that HEM and KO mice and cells display specific alterations in oxidative stress, DNA damage and expression of key pro-inflammatory cytokines that may contribute to the different tumor phenotypes observed. Furthermore, we demonstrate that p53 affects the expression of the NF-κB inhibitor IκBα, leading to altered NF-κB-regulated gene expression by promoting recruitment of p65 into *Nfkbia* promoter. These results reveal a novel mechanism of crosstalk between p53 and NF-κB transcription factors with impact on inflammation and cancer.

## Materials and methods

### Mice

The Trp53tm1Tyj mice^19^ were backcrossed to C57BL/6N background. P53 WT, p53 hemizygous (*Trp53*^*+/−*^, HEM) and p53 knockout (*Trp53*^*−/−*^, KO) mice were housed and bred in the Parc Científic of Barcelona and the Bellvitge’s animal facilities of the University of Barcelona. All mice procedures and methods were approved by the Ethics Committee for Animal Experimentation of the Catalan Government (# 7067 & 9119), and were performed in accordance with the relevant guidelines and regulations. Survival and spontaneous tumor development were monitored for up to 3 years. Mice were closely monitored and euthanized when they reached humane endpoint and autopsies were performed on these mice and the rest of the mice when they reached the 36 months. Tissues were fixed in 10% neutral-buffered formalin solution, processed for paraffin embedding and H&E staining and examined by a pathologist. Tumorigenic and leukocyte analysis were undertaken in 4-month-old male and female mice. A method of randomization was not used to determine how animals were allocated to each group. Pathologist was blinded to the mice genotype. Primers used to determine *Trp53* mouse genotypes by PCR were as follows: 5′TGGTTTGTGCGTCTTAGAGACAGT, 5′AAGGATAGGTCGGCGGTTCAT and CCAGCTCATTCCTCCCACTCA.

### Tissues, cell cultures, and treatment

Tissues were collected from WT, HEM, and KO mice and primary bone-marrow-derived macrophages (BMDMs) were prepared from bone marrow cells of the hind limbs of mice, as described^31^. Macrophages were obtained growing cells in Dulbecco’s Modified Eagle’s Medium supplemented with 20% FBS and 30% L-cell (medium obtained from the supernatant of L929 cells used as source of M-CSF, Macrophage-Colony Stimulating Factor*)* for up to 7 days. Then, cells were scrapped and seeded in plates for experiments. Cells were treated with 100 ng/ml LPS (Lipopolysaccharide) [Sigma] and 1 ng/ml IFNγ [ImmunoTools] for the indicated times. Cells were exposed to ultraviolet B (UVB) irradiation once medium was removed using a UV lamp [UVP Inc.] at the distance of 50 cm, and then fresh medium was added. A Radiometer with UVX-31 sensors was used [UVP Inc] to monitor UVB doses. The doses were 50 and 100 J/m^2^ for UVB.

### RT-qPCR analysis

Total RNA isolated from cells [NucleoSpin RNA extraction kit, Macherey-Nagel] was used for cDNA synthesis [GeneAmp RNA PCR kit, Applied Biosystems]. Quantitative PCR amplification reactions were performed in the 7500 Fast Real-Time PCR System [Applied Biosystems] using TaqMan Gene Expression Master Mix. Values were normalized to *Sdha*. The following TaqMan assays [Applied Biosystems] were used to quantify mRNA expression of mouse *Sdha* (Mm01352366_m1), *Il-1β* (Mm00434228_m1), *Il-1α* (Mm00439620_m1), *Mpo* (Mm01298424_m1) and *Tnfα* (Mm00443258_m1). The following primers were used to quantify mRNA expression: *Il-6* (Forward (F), AGTTGCCTTCTTGGGACTGA; Reverse (R), TCCACGATTTCCCAGAGAAC), *IL-12a* (F, GTACCAGACAGAGTTCCAGG; R, CGCAGAGTCTCGCCATTATG), *IL-18* (F, CTACCCTCTCCTGTAAGAAC; R, CTTGTTGTGTCCTGGAACAC), *IL-23a* (F, CAAGGACTCAAGGACAACAG; R, GGTGTGAAGTTGCTCCATG), *NFKbia* (F, AACCTGCAGCAGACTCCACT; R, GACACGTGTGGCCATTGTAG), *Trp53* (F, CACAACTGCACAGGGCAC; R, CATGGAGGAGTCACAGTCGG).

### Protein extracts and western blot analysis

Whole-cell extracts for western blotting were obtained in ice-cold lysis buffer containing 1% Nonidet P-40, 1% deoxycolate, 0.1% SDS, 50 mmol/L HEPES pH 7.5 and 150 mmol/L NaCl together with protease and phosphatase inhibitors (10 µg/ml aprotinin, 10 µg/ml leupeptin, 86 µg/ml iodoacetamide, 1 mM PMSF and 1 mM Na_3_VO_4_). For soluble and chromatin separations, cells were lysed in 1 mM EDTA, 0.1 mM Na-orthovanadate (Na_3_VO_4_), 0.5% Triton X-100, 20 mM β-glycerol-phosphate, 0.2 mM PMSF, protease inhibitor cocktail, in PBS for 20 min on ice and centrifuged at 13,000 rpm. Supernatants were recovered as the soluble fraction, and the pellets were lysed in Laemmli buffer (1× SDS-PAGE buffer plus β-mercaptoethanol [Sigma, Ref. M-3148]). For the analysis of proteins in cell supernatants, media of treated cells were collected, and liquids were evaporated using Speed Vaccum concentrator [Thermo Scientific]. Samples were boiled in Laemmli buffer and separated on SDS-PAGE. Western blot was performed as previously described^32^. Primary antibodies used throughout this study include, phospho-ATM (s1981) [Millipore, 05-740], ATM [Santa Cruz, sc-135663], β-actin [GenScript, A00702], phospho-Chk1 [Cell Signaling, #2348], Chk1 [Cell Signaling, #2360], COX2 [Cayman Chemical, 160116], phospho-Histone H2A.X (Ser 139) [Cell signaling, #2577S], H3 [Abcam, ab1791], IL-1β [Pierce, MM425B], IκBα [Santa Cruz, sc-371], IκBα phosphorylated in Ser32/36 [Cell Signaling, #9246], iNOS [Santa Cruz, sc-650], p53 [Cell Signaling, #2524] and tubulin [Sigma Aldrich, T6074]. Horseradish peroxidase-conjugated goat anti-rabbit or goat anti-mouse antibodies (1:10,000) [BioRad] were used as secondary antibodies. Detection was performed with enhanced chemiluminescence [Biological Industries] and autoradiography films [GE Healthcare]. Band quantification was performed using Adobe Photoshop CC 2015.

### Immunofluorescence analysis

Cells were fixed with 4% paraformaldehyde in PBS and permeabilized with 0.1% Triton X-100 in PBS. After blocking (20% FBS in PBS), samples were incubated with rabbit anti-p65 [Cell Signaling]. Goat anti-rabbit IgG coupled to Alexa Fluor 555 or 488 (1:500) was used as secondary antibody [Life Technologies]. Nuclei were stained with DAPI [Sigma]. Slides were mounted in ProLong Gold Anti-Fade reagent [Life Technologies]. Images of LPS-treated BMDMs were captured in an SP5 upright confocal microscope (Leica). Images of UVB-treated BMDMs were captured in an Eclipse E-800 Nikon fluorescence microscope.

### Myeloperoxidase activity

The activity of myeloperoxidase (MPO) was determined biochemically, as described previously^15^. First, the erythrocytes were lysed [Lysis buffer BD FACS, Becton, Dickinson *and Company*]. To release MPO from cells, the samples were homogenized in 0.5% hexadecyltrimethylammonium bromide in 50 mM potassium phosphate buffer, pH 6.0. After that, samples were subjected to three cycles of sonication, freezing and thawing. Then, they were centrifuged for 30 min at 14,000 rpm at 4 °C. The supernatants were mix with 0.334 mg/mL O-dianisidine dihydrochloride and 0.005% H_2_O_2_ in 50 mM potassium phosphate buffer, pH 6.0. In this enzymatic reaction H_2_O_2_ acts as substrate and O-dianisidine dihydrochloride as colorimetric reactive. MPO activity was measured spectrophotometrically over a 10 min period at 405 nm with a programmable microplate reader [Molecular Devices, Menlo Park, CA]. Data were analyzed with GraphPad Prism6 software.

### Flow cytometry analysis

Cells from the blood, spleen and thymus of 4-month-old *Trp53*^*−/−*^*, Trp53*^−^^*/+*^, and wt mice were stained following standard protocols with the following antibodies to lymphocyte cell surface markers: FITC anti-mouse CD3e [ImmunoTools, clone 145-2C11], PE anti-mouse CD4 [Immunotools, clone YTS 191.1.2], APC anti-mouse CD8 [Immunotools, clone YTS 169.4], FITC anti-mouse α/βTCR [Immunotools, clone H57-597], PE anti-mouse γδTCR [Immunotools, clone GL-3], FITC anti-mouse CD5 (BD Pharmigen, 553020) and PE anti-mouse CD19 [ImmunoTools, clone PeCa1]. Forward scatter versus side scatter plot was used to gate lymphocyte, monocyte, and granulocyte blood cells. Analysis were performed with a FACSCanto flow cytometer [Becton Dickinson].

### Oxidative stress analysis

Reactive oxygen species (ROS) levels were determined by incubating mouse blood 30 min at 37 °C with 5 μM CellROX Deep Red Reagent [Life Technologies], which emits fluorescence upon oxidation by ROS. After the staining, Red blood cells Ammonium Chloride lysis buffer was used (155 mM NH_4_Cl, 1 mM KHCO_3_, 0.1 mM EDTA in water, pH7.2). Fluorescence was measured with a FACSCanto flow cytometry [Becton Dickinson].

### Chromatin immunoprecipitation (ChIP)

Cells were crosslinked 10 min with 1% formaldehyde, lysed and sonicated. Chromatin fractions were incubated for 16 h with anti-p53 [abcam ab1101], anti-p65 [Abcam ab16502], mouse IgG [Sigma I5006] or rabbit IgG [Sigma I5381] antibodies in RIPA buffer and then precipitated with protein A/G-sepharose [GE Healthcare, Refs. 17-0618-01 and 17-0780-01]. Crosslinkage was reversed and DNA was purified. Then, qPCR was performed as explained above. Results were normalized to Input and Ig values. The following primers were used: p53 motif in Nfkbia promoter (F, GAGGCTAGCCACCACAGAAG; R, CTGGCTTGGGTCAATTGTTT), p53 motif in Mdm2 gene (F, GCCGAGTTGACTCAGCTCTT; R, TAAACGCTGGCAGGGGATTT), p65 motif in Nfkbia promoter (R, AGGCTGCAGGGAAGTACCTA; F, TAAACGCTGGCAGGGGATTT).

### Knockdown assays

Lentiviral particles containing two different MISSION shRNAs for mouse p53 [Sigma, TRCN0000310844 and TRCN0000331409] and non-target shRNA control [Sigma, SHC016] were obtained by transfection of plasmids into HEK293T cells, following standard procedures. One day after transfection, media was refreshed. Virus was collected 24 h later and then concentrated using Ultracentrifuge Optima™ XPN-100-IVD (Biosafe) [Beckman Coulter]. BMDMs were infected and after 24 h selected with puromycin (0.25 μg/ml) for 3 days before stimuli treatment.

### Statistical analysis

Statistical parameters, including number of events quantified, standard error of the mean (SEM), and statistical significance are reported in the figures and in the figure legends. Statistical analysis has been performed using GraphPad Prism6TM Software and *p* < 0.05 is considered significant. Two-sided Student’s *t* test was used to compare differences between genotypes or treatment conditions. Outliers were identified with Outlayer ROUT method (*Q* = 1%) using GraphPad Prism6TM.

## Results

### Specific pro-tumorigenic effects and hematopoietic alterations after *Trp53* single and double allele loss

**Fig. 1:**
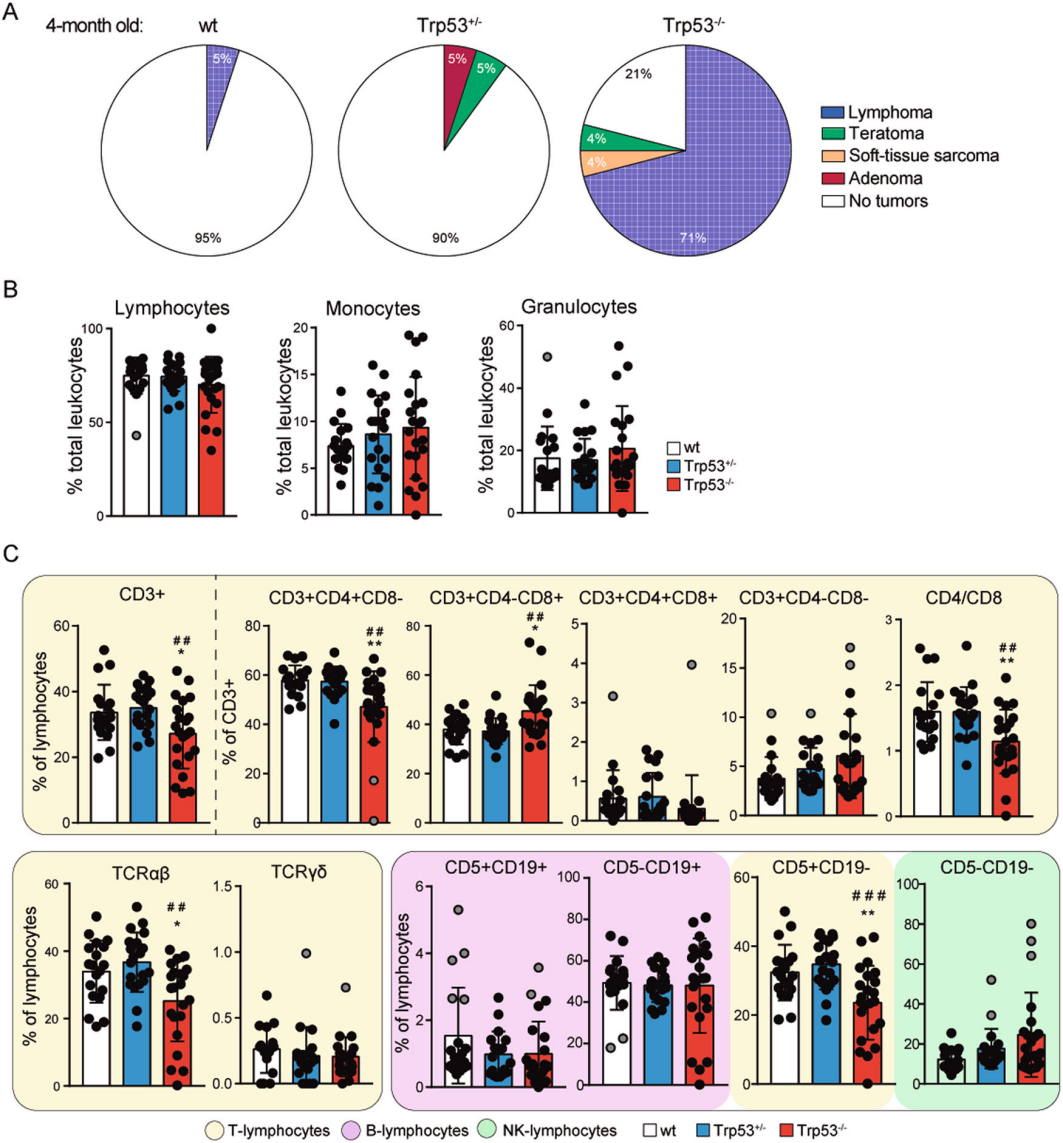
Tumor spectrum and lymphocyte subsets in p53 hemizygous and p53 knockout mice. **A** Percentage of tumor incidence and spectrum in 4-month-old WT, p53 hemizygous (HEM) and knockout (KO) mice. **B** Percentage of lymphocyte, monocyte, and granulocyte populations in bloodstream of 4-month-old WT, HEM and KO mice measured by flow cytometry analysis. **C** Percentage of lymphocyte populations in bloodstream of 4-month-old WT, HEM and KO mice measured by flow cytometry analysis. In **A**, **B** and **C**, at least 20 mice per genotype were analyzed. In **B** and **C**, bars represent mean values ± SEM. Significant differences and p values were derived from an unpaired *t*-test, two-tailed. WT vs HEM or KO: **p value < 0.005, *p value < 0.05. HEM vs KO: ^###^*p* value < 0.0005, ^##^*p* value < 0.005. Identified outliers were represented as gray dots.

We first analyzed the tumor incidence and spectrum and hematopoiesis of our p53-deficient mice (HEM and KO) maintained in a C57BL/6N background. Pathological analysis of mice at 4 months of age demonstrated that most p53 KOs (about 70%) developed early lymphomas in contrast with only few HEM animals (about 10%) developing adenomas and teratomas, and WT animals (about 5%) that developed lymphomas (Fig. 1A). At this relatively early age, all animals bared only one tumor type, matching percentages of tumor incidence with those of tumor spectrum. In agreement with this early phenotype, at the time of death, single and double p53 allele loss led to p53 dose-dependent decrease in survival (Fig. S1A), whereas tumor incidence is increased (Fig. S1B), and to a differential tumor spectrum (Fig. S1C), as described previously^15,17–24^. Notably, tumor spectrum at 4 months anticipated the tumor spectrum at time of death, showing a higher frequency of lymphomas in p53 KO than in HEM and WT mice. The early low lymphoma frequency seen in WT animals at 4 months of age as well as the predominance of lymphoma in those WT animals bearing tumors at time of death, are consistent with the fact that lymphoma are the most common tumors in many strains of mice, up to 20–40% in C57BL/6 strains^33^, including young individuals. Because lymphomas are the most frequent type of cancer in p53 KO mice, we studied the hematopoietic component. By flow cytometry analysis of peripheral blood, in 4-month-old mice we found comparable percentages of lymphocytes, monocytes and granulocytes in all three genotypes (Fig. 1B) but specific alterations in the lymphocyte subpopulations in the KOs (Fig. 1C). In particular, p53 KO mice showed a significant decrease in the percentage of total T cells (CD3+; CD5+ CD19−), mainly TCRαβ+ and T helper cells (CD3+ CD4+), and increased percentage of the T cytotoxic cells (CD3+ CD8+). Moreover, p53 KO mice showed increased percentage of NK cells (CD5-CD19−). No significant alterations in any of the analyzed hematopoietic cell populations were observed in WT and p53 HEM mice (Fig. 1C). Furthermore, the analysis of thymic populations showed obvious defects on thymocyte development only in the p53 KOs characterized by an increase in CD3+, single CD8+ and double CD4+ CD8+ subsets, but reduced numbers of single CD4+ cells and the CD4/CD8 ratio (Fig. S1D), which is in agreement with the changes in T lymphocytes observed in peripheral blood (Fig. 1C).

Together these results indicated that a single *Trp53* allele is sufficient to prevent early tumorigenesis that primarily arises in hematopoietic double deficient *Trp53* cells. However, p53 HEM mice still developed a reduced number of solid tumors including adenomas and teratomas.

### Loss of one *Trp53* allele leads to increased oxidative status in peripheral blood granulocytes and bone marrow-derived macrophages (BMDMs)

**Fig. 2:**
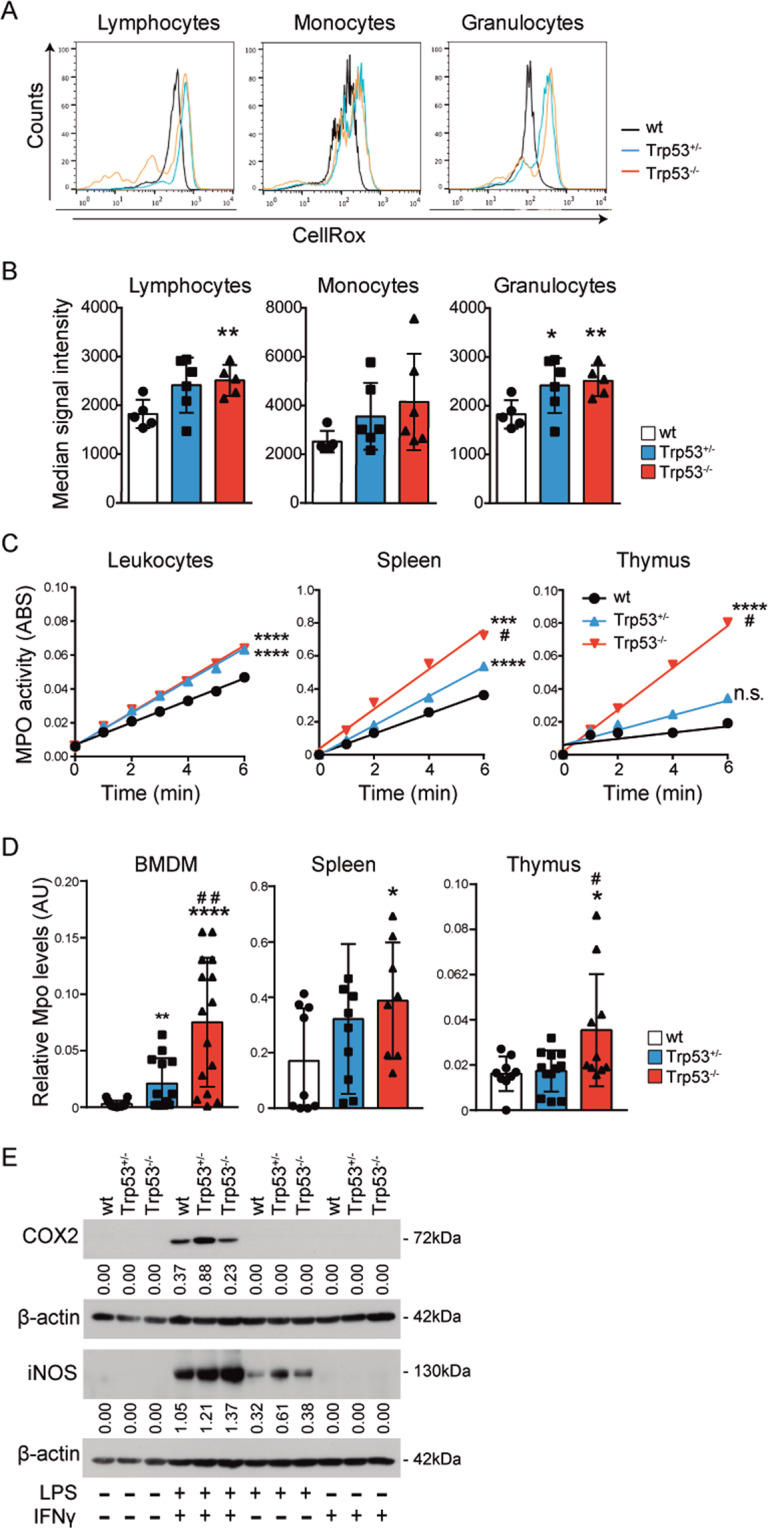
Loss of one *Trp53* allele leads to an increased oxidative status in immune cells and tissues. **A** Representative histograms showing oxidative stress analysis by flow cytometry using CellROX reagent in the major subsets of blood leukocyte samples: lymphocytes, monocytes, and granulocytes from 4-month-old p53 WT, HEM, and KO mice. **B** Quantification of CellROX by flow cytometry analysis. Five mice per genotype were analyzed. **C** Myeloperoxidase (MPO) activity analysis in peripheral blood leukocytes, spleen and thymus samples from 4-month-old WT, HEM, and KO mice. At least five mice per genotype were analyzed. Graphs represent the absorbance (ABS) of O-dianisidine dihydrochloride, which reflect the amount of H_2_O_2_ processed by MPO. **D** Relative mRNA expression of the Myeloperoxidase (*Mpo*) gene in bone marrow-derived macrophages (BMDMs), spleen and thymus from 4-month-old WT, HEM, and KO mice. For BMDM quantification, biological replicates were performed in five independent experiments. For hematopoietic tissues quantification, at least eight mice per genotype were analyzed. **E** Protein expression of ciclooxygenase 2 (COX2) and inducible nitric oxide synthase (iNOS) in BMDMs of the indicated genotypes treated for 24 h with LPS (100 ng/ml) and/or IFNγ (1 ng/ml). Relative expression values were referred to β-actin expression. Representative image of a western blot analysis from two independent experiments is shown. In **B** and **D**, bars represent mean values ± SEM. AU arbitrary units. Significant differences and *p* values were derived from an unpaired *t*-test, two-tailed. In **C**, linear regression analyses were performed. WT vs HEM or KO: ****p value < 0.0001, ***p value < 0.0005, **p value < 0.005, *p value < 0.05, n.s. non-significant. HEM vs KO: ^##^*p* value < 0.005, ^#^*p* value < 0.05.

Reactive Oxidative Species (ROS) are among the best-known triggers of carcinogenesis^34^ and multiple evidences support the important role of p53 in regulating intracellular redox homeostasis^34,35^. Thus, we speculated that differences in the oxidative stress might contribute to the differential tumorigenic phenotype of p53 HEM and KO mice. We used CellROX and flow cytometry to quantify the impact of p53 levels in ROS production from the major leukocyte subsets (lymphocytes, monocytes and granulocytes) (Fig. S2A, B). Analysis of tumor-free 4-month-old p53 WT, HEM and KO mice indicated that levels of ROS were higher in p53 KO granulocyte and lymphocytes than in the corresponding WT cell subsets. ROS levels were also elevated in p53 HEM cells, although significant statistical differences were only detected in the granulocyte component (Figs. 2A, S2B). To further investigate this phenotype, we analyzed the enzymatic activity of the MPO enzyme, a key player in the generation of ROS^36^, in peripheral blood leukocytes, spleen and thymus. MPO was mainly expressed in hematopoietic stem cells (HSCs), myeloid and granulocytic progenitors, granulocytes and natural killer (NK) cells (Fig. S2C). Our results demonstrated that p53 KO imposed a significant increase in MPO activity in circulating leukocytes, spleen and thymus cell populations, whereas loss of a single *Trp53* allele similarly affected circulating leukocytes and the spleen cells but had a minor effect on the thymus component (Fig. 2C). Analysis of *Mpo* mRNA expression in p53 WT, HEM and KO BMDMs, spleen and thymus cells closely correlated with MPO activity (Fig. 2D), indicating that MPO regulation can take place at least partially at the transcriptional level.

Inducible nitric oxide synthase (iNOS) and cyclooxygenase 2 (COX2) contribute to the generation of the nitric oxide (NO) and superoxide (O2^−^) reactive species, respectively, and are key pro-oxidant enzymes^37^. Western blot analysis of BMDMs indicated that upregulation of COX2 after treatment with LPS plus IFNγ was higher in the absence of one *Trp53* allele compared to WT or p53 KO cells (Fig. 2E). In a similar way, LPS alone induced higher expression of iNOS in p53 HEM than KO or wt BMDM, whereas stimulation with combined LPS and IFNγ induced higher levels than wt in both p53 HEM and KO BMDM. However, iNOS and COX2 activation following single or compound LPS and IFNγ treatment was determined at a single time point, thus results should not be considered as absolute as they integrate possible differences in activation kinetics between p53 genotypes.

Overall, reduction of p53 levels affects oxidative stress in different hematopoietic cell populations and tissues. The loss of one allele is sufficient to increase oxidative stress in granulocytes, BMDMs and spleen cells. However, single allele of *Trp53* precludes enhanced ROS production imposed by complete p53 deficiency in lymphocytes and thymus cells, which is in agreement with the higher incidence of lymphomas in p53 KO mice.

### Loss of one *Trp53* allele enhances the expression of pro-inflammatory cytokines in untreated and stress-treated BMDMs

**Fig. 3:**
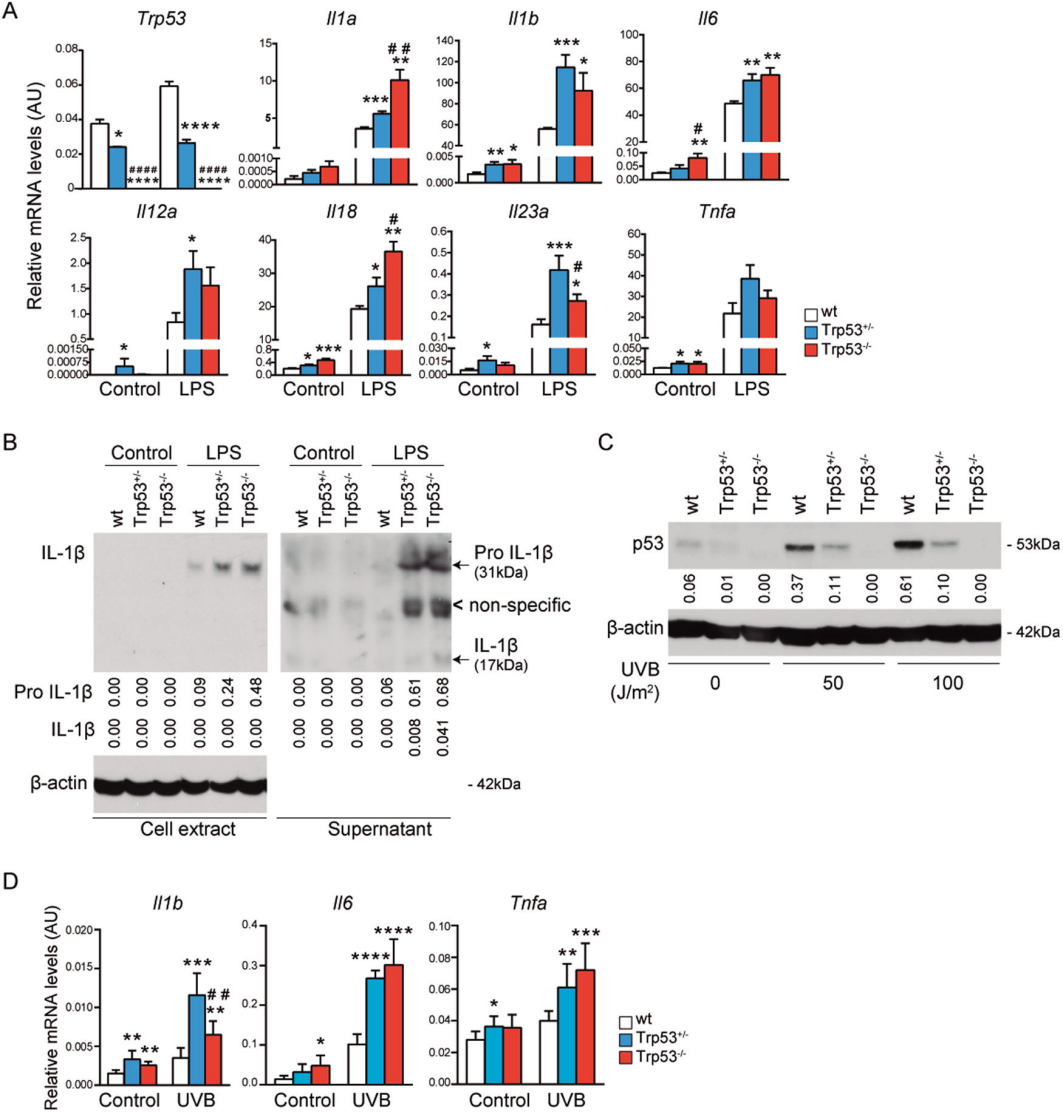
Loss of one *Trp53* allele leads to an increased pro-inflammatory cytokine expression in untreated and LPS and UVB-treated BMDM cells. **A** Relative mRNA expression of the indicated genes in BMDMs from p53 WT, HEM, and KO mice untreated (control) and treated (LPS, 100 ng/ml, 4 h). Biological replicates were quantified in four independent experiments. **B** Expression of IL-1β pro-form (31 kDa) and active isoform (17 kDa) in total cell extracts and supernatants of BMDMs from WT, HEM, and KO mice untreated (control) and treated (LPS, 100 ng/ml, 4 h). Relative expression values in cell extracts were referred to β-actin expression. Representative image of a western blot analysis from at least two independent experiments is shown. **C** Expression of p53 in BMDMs from WT, HEM, and KO mice untreated and irradiated with UVB (50 or 100 J/m^2^, 24 h). Relative expression values were referred to β-actin expression. Representative image of a western blot analysis from two independent experiments is shown. **D** Relative mRNA expression of the indicated pro-inflammatory cytokine genes. BMDMs from WT, HEM, and KO mice were untreated and treated with UVB (50 J/m^2^, 1 h). Biological replicates in three independent experiments were analyzed. In **A** and **D**, bars represent mean values ± SEM. AU arbitrary units. Significant differences and *p* values were derived from an unpaired *t*-test, two-tailed. WT vs HEM or KO: ****p value < 0.0001, ***p value < 0.0005, **p value < 0.005, *p value < 0.05. HEM vs KO: ^####^*p* value < 0.0001, ^##^*p* value < 0.005, #*p* value < 0.05.

Oxidative stress response is frequently associated with the pro-inflammatory response^38–41^ and p53 deficiency has been linked with increased expression of pro-inflammatory cytokines^9–11^. To determine whether single *Trp53* allele loss influenced the inflammatory response, we performed RT-qPCR analysis of several key pro-inflammatory cytokines in BMDMs. As expected, p53 KO cells did not express *Trp53* mRNA and p53 HEM cells expressed approximately 50% compared with WT cells, and this proportion was maintained after LPS treatment (Fig. 3A). Interestingly, p53 KO and HEM BMDMs showed increased mRNA expression of the cytokines IL-1β, IL-6, IL-12α, IL-18, IL-23α, and TNFα in the absence of LPS stimulation. Of note, LPS-induced mRNA expression of the studied cytokines was higher in p53 HEM and KO BMDMs than in WT cells. Interestingly, *Il-23α* expression was significantly higher in the HEM than in KO cells whereas the expression of *Il-1α* and *Il-18* was higher in KO than HEM cells (Fig. 3A). Consistent with the *Il-1β* mRNA expression data, protein levels of IL-1β (31 kDa pro-form and 17 kDa active form) were increased in LPS-treated p53 HEM and KO BMDMs compared with the WT (Fig. 3B). The active form was higher in KO than in HEM cells, suggesting a higher activation of the inflammasome^42^ by p53 dosage.

To analyze whether p53 levels affected the immune response to other stress stimulus, we checked the response to UVB radiation. Importantly, UVB treatment induced a dose-dependent stabilization of p53 protein, as described previously^43–46^, which was significantly lower in HEM cells than in WT (Fig. 3C). Similarly to LPS response, UVB-induced expression of the main pro-inflammatory cytokines IL-1β, IL-6, and TNFα was higher in p53 HEM and KO BMDMs compared to WT (Fig. 3D).

Overall, these results indicate that the loss of a single *Trp53* allele is sufficient to trigger a pro-inflammatory response in homeostasis as well as increase the stress-induced expression of several key pro-inflammatory cytokines. Furthermore, *Trp53* allele loss could affect kinetics of expression, as previously mentioned.

### Increased nuclear p65/NF-κB localization in stress-treated BMDM cells by the loss of a single *Trp53* allele

**Fig. 4:**
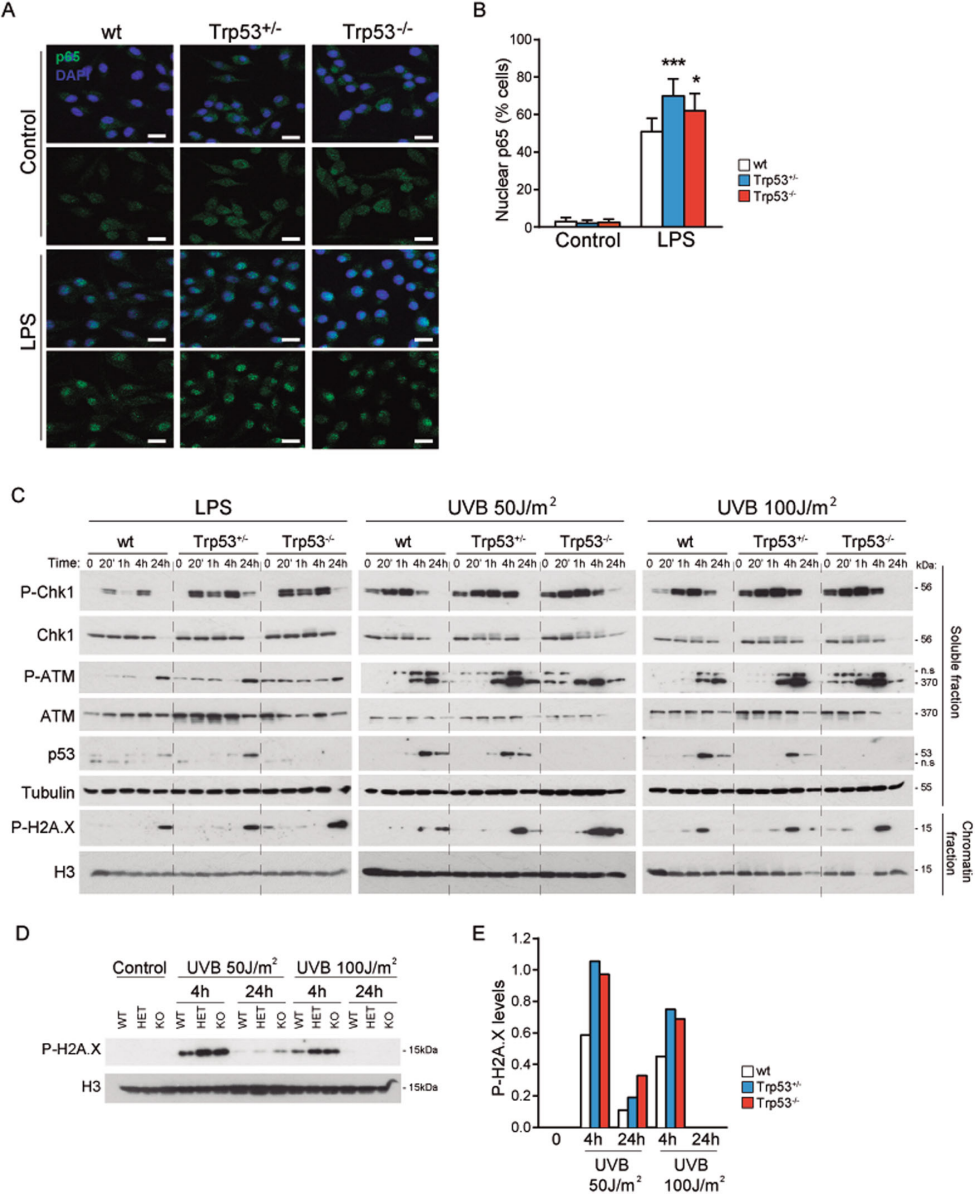
Loss of one *Trp53* allele leads to increased p65/NF-κB nuclear localization and increased DNA damage response in LPS- and UVB-treated BMDM cells. **A** Representative p65 immunofluorescence images of untreated (Control) and treated (LPS, 100 ng/ml, 15 min) BMDMs from p53 WT, HEM and KO mice. **B** Quantification of the percentage (%) of cells positive for nuclear p65 localization in control and LPS (100 ng/ml, 15 min)-treated BMDMs. A minimum of 500 cells per genotype was counted in two independent experiments. **C** Expression of DNA damage response proteins in BMDMs treated with LPS (100 ng/ml) or UVB (50 J/m^2^ and 100 J/m^2^) for 4 or 24 h (h) from p53 WT, HEM, and KO mice. Representative images of western blot analysis from two independent experiments are shown. n.s. non-specific band. **D** Expression of P-H2A.X in BMDMs treated with UVB (50 J/m^2^ and 100 J/m^2^) for 4 or 24 h (h) from p53 WT, HEM, and KO mice. Representative images of western blot analysis from two independent experiments are shown. **E** Quantification of western blot analysis (in **D**) of UVB-treated WT, HEM, and KO BMDMs. Means of two independent experiments are shown. In **A**, scale bars are 25 μm. In **B**, bars represent mean values ± SEM. AU arbitrary units. Significant differences and *p* values derived from an unpaired *t*-test, two-tailed. WT vs HEM or KO: ***p value < 0.0005, *p value < 0.05.

Induced expression of pro-inflammatory cytokines lays mainly downstream of NF-κB signaling^47^. Thus, we next studied whether increased cytokine expression in p53 HEM and KO cells was due to increased or sustained p65/NF-κB signaling. By immunofluorescence analysis we failed to detect any nuclear (active) p65/NF-κB in untreated cells of either genotype. Nevertheless, nuclear p65/NF-κB translocation after LPS (Fig. 4A, B) or UVB (Fig. S3A, B) treatment was significantly higher in p53 HEM and KO cells than in WT BMDMs. These results indicated that the loss of *Trp53* alleles impose a dose-dependent effect on LPS- and UVB-induced activation of key pro-inflammatory cytokines, associated with increased p65/NF-κB signaling.

### Increased DNA damage in stress-treated BMDM cells by the loss of a single *Trp53* allele

As inflammation and pro-inflammatory response, DNA damage and the DNA damage response were specifically affected by the single and double loss of *Trp53* alleles (Figs. 4C, S4A). In particular, the DNA repair signaling pathways to single-strand breaks (SSB, assessed by levels and kinetics of P-Chk1) as well as double-strand breaks (DSB, assessed by levels and kinetics of P-ATM) were induced in a particular way by the loss of single and double *Trp53* allele compared to WT after LPS and UVB threats (Figs. 4C and S4A). Higher amount of the P-H2A.X, mark of DNA strand breaks^48^, accumulated in KO and HEM compared to WT cells after UVB stimuli. Intriguingly, P-H2AX levels were slightly stronger and lasted longer in UVB-treated cells at low (50 J/m^2^) than at high (100 J/m^2^) doses (Fig. 4C–E). This fact could be in part related to a higher rate of cell death at high than low UVB doses. Also, it cannot be ruled out a relatively highest peak of DNA damage in the high dose UVB-treated cells between 1 and 4 h. On the other hand, KO cells but not HEM cells did show high levels of DNA damage than WT cells at 24 h after LPS treatment. Consistently, stabilization of p53 protein, which followed similar kinetic patterns in WT and HEM cells, peaks at 24 h after LPS and 4 h after UVB (Figs. 4C, S4A). The earlier and stronger DNA damage response upon UVB radiation compared to LPS treatment would be related to the direct DNA damaging activity of UVB in contrast with the indirect oxidative DNA damage triggered by LPS as consequence of the production of ROS, among other reactive species. Overall, these results indicate that single *Trp53* allele loss results in an increase of DNA damage upon genotoxic stimuli, compromising thus genomic stability.

### Increased NF-κB signaling and delayed IκBα re-synthesis by the loss of a single *Trp53* allele

**Fig. 5:**
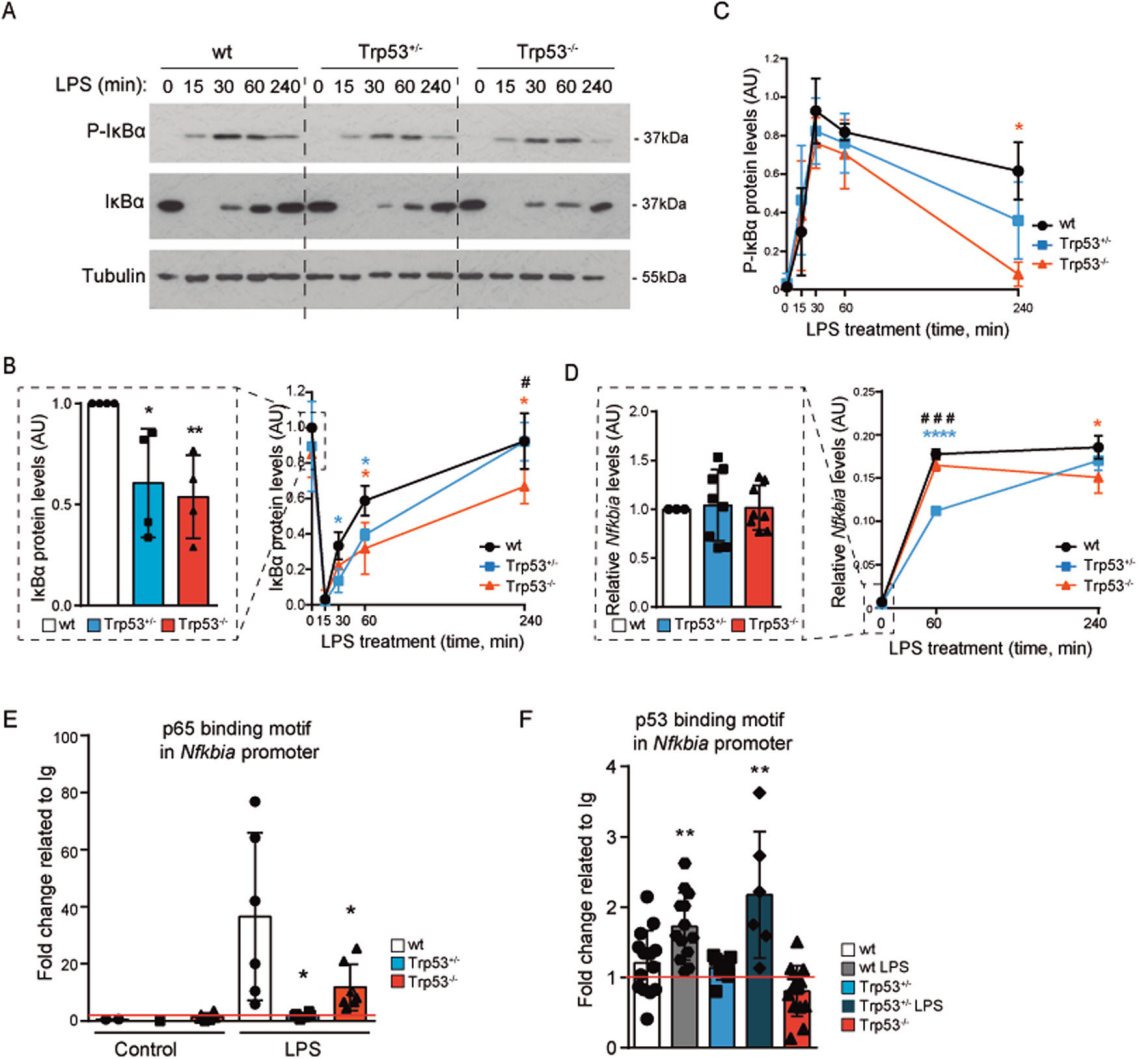
Loss of one *Trp53* allele affects NF-κB pathway via regulation of IκBα mRNA expression. **A** Levels of P-IκBα and IκBα in untreated and LPS-treated (100 ng/ml) BMDMs from p53 WT, HEM and KO mice at the indicated times. Representative image of a western blot analysis from three independent experiments is shown. **B** Quantification of IκBα levels from three independent western blot analysis. A graph for basal levels (left panel) and a graph for LPS treatment (right panel) are shown. Expression values were referred to tubulin levels. **C** Quantification of P-IκBα levels from three independent western blot analysis. Expression values were referred to tubulin levels. **D** Relative expression of *Nfkbia* mRNA in BMDMs from WT, HEM and KO mice treated with LPS (100 ng/ml) at the indicated times. A graph for basal levels (left panel) and a graph for LPS treatment (right panel) are shown. Values refer to biological replicates in three independent experiments. **E** p65 ChIP qPCR of *Nfkbia* promoter. WT, HEM, and KO BMDMs were untreated and treated with LPS (100 ng/ml, 60 min) before ChIP was performed. Values were normalized to input and the results are shown as fold change related to control immunoglobulin (Ig) sample. Values refer to biological replicates of two independent experiments. **F** p53 ChIP qPCR of *Nfkbia* promoter. BMDMs were untreated or treated with LPS (100 ng/ml, 60 min) before ChIP was performed. Values are normalized to input and the results are shown as fold change related to Ig sample. Values refer to biological replicates of three independent experiments for WT and KO, and one for HEM BMDMs. In **B**, **D**, **E** and **F**, bars represent mean values ± SEM. In **B**, **C** and **D** (right panels) mean values ± SEM are represented. AU arbitrary units. Significant differences and *p* values were derived from an unpaired *t*-test, two-tailed. wt vs HEM or KO. ****p value < 0.0001, **p value < 0.005, *p value < 0.05. HEM vs KO. ^###^*p* value < 0.0005, ^#^*p* value < 0.05.

To further investigate the mechanisms of NF-κB miss-regulation imposed by single *Trp53* allele loss, we measured the levels and phosphorylation of IκBα, which is the main inhibitor of NF-κB signaling^49^. We detected lower levels of IκBα in p53 HEM and KO BMDMs compared with WT cells (Fig. 5A, B), consistent with higher basal cytokine production in p53 HEM and KO cells. The non-overlapping IκBα degradation and phosphorylation kinetics seen after LPS treatment (Fig. 5A) is in agreement with the oscillation dynamics of NF-kB signaling^50^. Most importantly, recovery of IκBα protein levels after LPS-induced IκBα phosphorylation and subsequent degradation, which is required for post-activation repression of NF-κB signaling^51,52^, was significantly reduced in p53 HEM and KO cells (Fig. 5A, B). However, we detected comparable levels of P-IκBα in WT, HEM, and KO cells after LPS treatment (Fig. 5A, C) thus suggesting that delayed protein recovery in p53 HEM and KO cells was not due to the increased IκBα degradation but instead of defective LPS-induced transcription of the *Nfkbia* gene. In agreement with this hypothesis, we detected significant lower *Nfkbia* levels in p53 HEM and KO BMDM cells following LPS treatment (Fig. 5D), suggesting that p53 might regulate transcription of the *Nfkbia* gene, a known target gene of p65 transcription factor^52^.

Synergistic effects of p53 and p65 on *Nfkbia* gene expression have been previously suggested^53^. To further explore the potential mechanism by which p53 transcriptional activity might control NF-κB pathway, we performed in silico study of the *Nfkbia* promoter region. The analysis revealed the presence of binding consensus sites for p53 as well as p65 (Fig. S5A). p65 ChIP experiments in LPS-treated BMDMs indicated that p53 HEM and KO cells showed significantly less p65 binding to *Nfkbia* promoter (Fig. 5E), which correlates with reduced LPS-induced IκBα mRNA levels (Fig. 5D). p53 ChIP data showed that upon LPS stimulation p53 binds the promoter region of *Nfkbia* in WT and HEM cells (Fig. 5F). As described^54^, we did observe p53 binding to the intronic region of Mdm2 upon UVB (Fig. S5B, C), and as expected, no p53 binding was detected when using p53 KO cells (Figs. 5F, S5C).

Overall, these results indicate that p53 influences NF-κB signaling by transcriptional regulation of the NF-κB inhibitor IκBα, uncovering a novel crosstalk mechanism between the p53 and NF-κB transcription factors. Hence, p53 dosage might impose differential amounts, durations and patterns of cytokine expression with great impact on inflammation, oxidative stress, and tumorigenesis.

### Increased NF-κB signaling and enhanced expression of pro-inflammatory cytokines by p53 knockdown

**Fig. 6:**
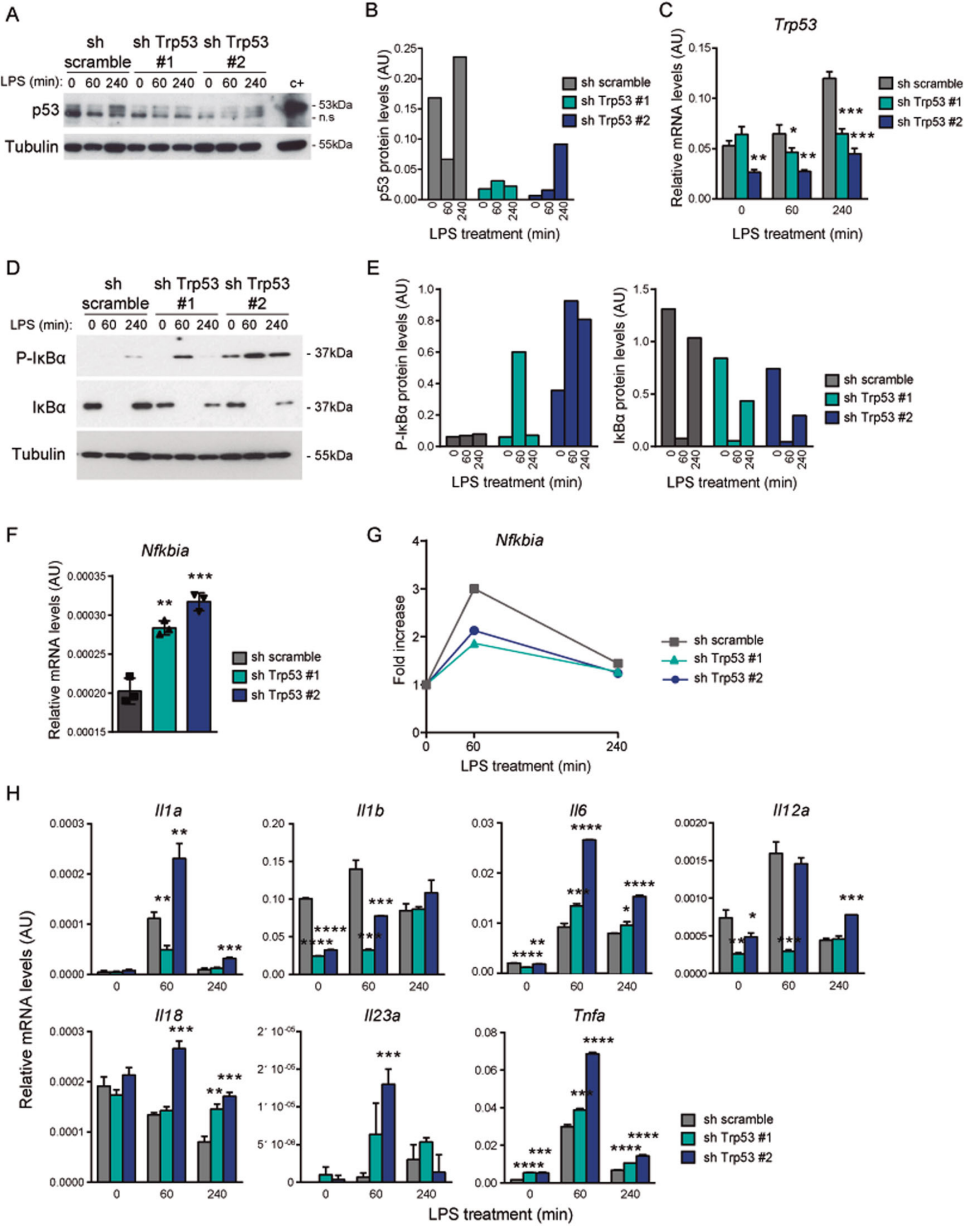
p53 knockdown leads to an increased pro-inflammatory cytokine expression and affects NF-κB pathway. WT BMDMs infected with sh scramble, sh *Trp53* #1 or sh *Trp53* #2 were untreated and treated with LPS (100 ng/ml) for the indicated times. **A** p53 protein expression and **B** quantification. Representative images of western blot analysis from one experiment are shown. C+, positive control for p53 staining from UVB-treated cells. Relative expression values were referred to tubulin expression. **C** Relative mRNA expression of *Trp53*. Technical replicates were quantified in one experiment. **D** P-IκBα and IκBα protein expression and **E** quantification. Representative image of a western blot analysis from one experiment is shown. Relative expression values were referred to tubulin expression. **F** Relative mRNA expression of *Nfkbia* in untreated BMDMs. Technical replicates were quantified in one experiment. **G** Relative mRNA expression of *Nfkbia* in LPS-treated BMDMs. Technical replicates were quantified in one experiment. **H** Relative mRNA expression of the indicated genes. Technical replicates were quantified in one experiment. AU arbitrary units. In **C**, **F**, **G** and **H** bars represent mean values ± SEM. Significant differences and *p* values were derived from an unpaired *t*-test, two-tailed. Sh scramble vs sh *Trp53* #1 or sh *Trp53* #2. ****p value < 0.0001, ***p value < 0.0005, **p value < 0.005, *p value < 0.05.

To further demonstrate the impact of *Trp53* dosage in the pro-inflammatory response, p53 expression was knockdown (KD) in WT BMDM cells by means of shRNA lentivirus infection. Two shRNA tested against *Trp53* (#1 and #2) led to significant decrease in the levels of p53 protein (Fig. 6A, B) and RNA (Fig. 6C) compared to shRNA scramble. In agreement with the differential inflammatory response triggered by *Trp53* allele loss, the knockdown of p53 led to an overactivation of NF-κB pathway after LPS stimulation. Thus, the levels of P-IκBα were increased in parallel with the decrease in the levels of total IκBα (Fig. 6D, E) in shRNA *Trp53*-treated cells compared with shRNA scramble-treated cells. This effect was also observed in non-stimulated cells. This basal activation of NF-κB in KD cells also induce an overexpression of *Nfkbia* (Fig. 6F), since this gene is one of the main NF-κB target^52^. However, as seen in HEM and KO cells, the decreased levels of p53 avoid the proper expression of *Nfkbia* after LPS stimulation (Fig. 6G). Consequently, levels of p53 KD and the overactivation of NF-κB pathway upon LPS correlated with an enhanced mRNA expression of the pro-inflammatory cytokines IL-1α, IL-6, IL-18, and TNFα (Fig. 6H). Although the same effect was not observed with IL-1β, when the fold change of expression related to un-stimulated condition is analyzed, we observed a significant increased expression in LPS-treated KD BMDMs (Fig. S4B). Therefore, differences in the expression and kinetics of cytokines might be related to the different KD efficiency of the two tested *Trp53* shRNA. Overall, these results corroborate that a decrease in p53 levels, both by single *Trp53* allele loss or p53 KD, drive an increase in the inflammatory response mediated at least partially by activation of NF-κB pathway.

## Discussion

The pivotal role of p53 in maintaining genome integrity to avoid tumorigenesis has been extensively studied, including its role in controlling cell cycle, apoptosis, cellular stress and the inflammatory response, among other functions. Moreover, the importance of p53 dosage in cancer has become unequivocally evident in mice carrying supernumerary copies of the *Trp53* gene^55,56^ and in mice with single or double germline deletion of *Trp53* alleles that show a very different incidence and type of tumors relative to WT mice^15,17–24^, as in the present study. In fact, tumor spectrum and incidence in p53 HEM mice, rather that KO mice, are more similar to those found in individuals with Li-Fraumeni syndrome, associated with germline null or inactivating genetic mutations in a single allele of the *TP53* gene^16,57–60^. Of note, ~20% of these patients and 50% of the p53 heterozygous tumors still retain the wt allele of the gene^16,23,61^, indicating that p53 haploinsufficiency by loss of one *Trp53* allele can be sufficient to promote carcinogenesis. Nevertheless, the reasons and mechanisms remain poorly known. In this context, our work provides novel insights uncovering manifest differences between mice carrying germline deletion of one (HEM) or both (KO) *Trp53* alleles in oxidative, DNA damage and inflammatory responses, well-known triggers of carcinogenesis that, at least in part, could underlie their distinct tumor phenotype. In this manner, the single *Trp53* allele loss compromises the maintenance of genomic stability, although to less extension than the complete loss, mainly in the response to primary DNA damaging agents (e.g., UVB) rather than to secondarily induced DNA damage by stress threats (e.g., LPS)^62^. Notably, our data also indicate that the loss of one *Trp53* allele directly affects IκBα expression, which leads to altered NF-κB activation and thereby altered pattern and levels of pro-inflammatory cytokine expression, a key non-cell autonomous mechanism that influences both tumor progression and suppression^63^.

Relevantly, the differences between single and double loss of *Trp53* alleles in the oxidative status in hematopoietic cells and tissues closely parallels the distinct frequency and type of lymphomas reported in p53 HEM and KO mice. Hence, the low frequency of thymic lymphomas and predominance of splenic lymphoma in HEM mice^20,21,23^ correlates well with the fact that HEM spleen but not thymus show an increase in oxidative stress markers. On the other hand, the highest frequency of lymphomas, especially thymomas, in KO mice^20,21,23^ parallels with an increased oxidative status in KO thymus and spleen. Noteworthy, the higher percentage of CD8+ T cells observed in the p53 KO mice was also observed in patients with thymomas^64^. These findings match with the increased oxidative status reported in blood and thymus of p53 KO mice^9^, human cancer cells upon downregulation of p53^65^ and, interestingly, blood and tissue samples from Li-Fraumeni syndrome individuals^66,67^. Therefore, mechanisms involved in the maintenance of redox homeostasis are among the molecular basis that can explain the p53 dosage-dependent differences in tumor spectrum.

In addition, our work reveals that reduced p53 dosage, resulting from either single and double germinal allele loss or knockdown, impacts on the oxidative and inflammatory status by upregulating differently the expression of key pro-oxidant genes and pro-inflammatory cytokines in macrophages, in either basal conditions and after stressor signals. For instance, in basal conditions, *Il6* is upregulated only in p53 KO BMDMs, whereas *Il-23a* is upregulated only in HEM BMDMs. In addition, LPS-induced expression of *Il1a* and *Il-18* is higher and *Il-23* is lower in KO than in HEM BMDMs. Several evidences indicate that the pattern and balance of pro-inflammatory cytokines, including IL-1α, IL-1β, IL-6, IL-12, IL-18, IL-23 and TNF-α, among others, play a crucial role shaping protumor and antitumor immunity and thereby the development of tumorigenesis^63^, as reflected in the differential tumor phenotype of p53 KO and HEM mice. Increasing evidences highlight p53 as a key player in immunity and inflammation by regulating directly or indirectly the expression of numerous key immune genes^68,69^. The present results support a role of p53 as repressor of NF-κB-dependent proinflammatory cytokine induction in mouse macrophages, which is in agreement with previous studies in p53-deficient mice^9,11,70,71^. Intriguingly, p53 stabilization by nutlin-3 leads also to an increase in the expression of proinflammatory cytokines^72^, pointing to the critical role of the levels of p53 expression in determining its function. Importantly, our data indicate that *Nfkbia* can be a direct target gene for p53 in the stress inflammatory response, unveiling a novel transcriptional co-activation of IκBα expression by p53 and p65. To our knowledge, this is the first time to identify a p53-binding site in the *Nfkbia* promoter. After stimulation, low or null levels of p53 will impair an efficient p65-dependent transcriptional re-expression of IκBα, which is needed to restrain the activation of the NF-κB pathway^51,52^. These may represent a new feedback control mechanism of p53 over NF-κB signaling pathway upon stress conditions and an additional link in the dense and complex connection nets taking place between the two transcription factors. In this context, direct and indirect mutual regulation^73,74^ and antagonistic^75–77^ or cooperative^53,72,74,78–80^ effects between p53 and NF-κB in transcriptional regulation depending of the gene, stimuli, cell type or physiological conditions have been largely documented.

In sum, the present work provides novel insights in the mechanisms and consequences of the loss of one *Trp53* allele, using p53 HEM mice as a model to study underlying pathomechanism of individuals with Li-Fraumeni syndrome. Single *Trp53* allele loss decreases more than half the amount of p53 protein, triggering an increased oxidative, DNA damage and inflammatory status by reducing the expression of IκBα, ultimately leading to enhanced tumorigenesis. The dependence of p65 on p53 for negative feedback regulation of NF-KB it is of great interest as it might potentially impact treatment strategies for patients suffering from autoinflammatory as well as cancer diseases. Further studies are yet needed to fully characterize in detail the complex crosstalk mechanisms between these two relevant transcription factors that link inflammation and tumorigenesis.

## Supplementary information

Supplementary Figure Legends

Supplemental Figure S1

Supplemental Figure S2

Supplemental Figure S3

Supplemental Figure S4

Supplemental Figure S5
