# Supplementary material for: Single loss of a Trp53 allele triggers an increased oxidative, DNA damage and cytokine inflammatory responses through deregulation of IκBα expression
Source: Cell Death Dis. 2021 Apr 6;12(4):359. doi: 10.1038/s41419-021-03638-3 (PMC8024389; doi:10.1038/s41419-021-03638-3)
Supplement: Supplementary file 1 — Supplementary Figure Legends [file 41419_2021_3638_MOESM1_ESM.pdf]

Supplementary figure legends

Figure S1. Survival, tumor spectrum and lymphocyte subsets in p53 HEM and KO mice. (A) Survival data of WT, HEM and KO mice. (B) Tumor incidence of WT, HEM and KO mice. (C) Tumor spectrum in WT, HEM and KO mice at the time of death. For survival, incidence and tumor development studies 24 WT, 51 HEM and 45 KO mice were analyzed. (D) Percentage of T-lymphocyte populations in the thymus of 4-month-old WT, HEM and KO mice. 13 WT, 4 HEM and 9 KO mice per genotype were analyzed. Bars represent mean values ± SEM. Significant differences and p-values were derived from an unpaired *t*-test, two-tailed. WT vs KO: ***p-value<0.0005, **p-value<0.005.

Figure S2. Oxidative stress analysis in blood leukocyte cells. (A) Representative flow cytometry SSC/FSC histogram analysis of the peripheral blood leukocyte subset (lymphocytes, monocytes and granulocytes) from p53 WT, HEM and KO mice. (B) Representative flow cytometry histogram images of CellROX fluorescence analysis in lymphocytes, monocytes and granulocytes, from WT, HEM and KO mice. (C) *Mpo* expression analysis in hematopoietic cell types obtained from previously published expression array (GSE55732) (ref. 81).

Figure S3. Loss of one *Trp53* allele leads to increased p65/NF-κB nuclear localization in UVB-treated BMDM cells. (A) Representative p65 immunofluorescence images of BMDMs from p53 WT, HEM and KO mice untreated (Control) and treated (UVB 100J/m^2, 1 hour). (B) Quantification of the percentage (%) of cells positive for nuclear p65 localization in control and UVB (100J/m^2, 1 hour)-treated BMDMs. A minimum of 500 cells per genotype was counted in two independent experiments. In Figure B, bars represent mean values ± SEM. AU, arbitrary units. Significant differences and p-values derived from an unpaired *t*-test, two-tailed. WT vs HEM or KO: **p-value<0.005, *p-value<0.05. HEM vs KO: # p-value<0.05.

Figure S4. Quantification of DNA damage response and *il1b* expression. (A) Quantification of Western Blot analysis (in Figure 4C) of LPS- and UVB-treated WT, HEM and KO BMDMs. Means of two independent experiments are showed. (B) Quantification of *il1b* expression in WT BMDMs infected with sh scramble, sh *Trp53* #1 or sh *Trp53* #2 shown in Figure 6H. Technical replicates were quantified in one experiment. Bars

represent mean values ± SEM. Significant differences and p-values were derived from an unpaired *t*-test, two-tailed. Sh scramble vs sh *Trp53* #1 or sh *Trp53* #2. ****p-value<0.0001, ***p-value<0.0005, **p-value<0.005. AU, arbitrary units.

Figure S5. p53 and p65 binding motifs in *Nfkbia* and *Mdm2* genes. (A) Representation of *Nfkbia* promoter, where the p53 and p65 binding motifs were represented. In the upper part, the consensus sequence was depicted and in the lowest part, the sequence present in the genome and the length of the amplified fragment. [n*]: nucleotides do not present in the consensus sequence (179 in p53 motif and 140 in p65 motif). (B) Representation of *Mdm2* gene, where the p53 binding motif was represented. In the upper part, the consensus sequence was depicted and in the lowest part, the sequence present in the genome and the length of the amplified fragment. E: exon. (C) p53 ChIP qPCR of *Mdm2* intronic region. UVB (50 J/m^2, 60 min). Values are normalized to input and the results are shown as fold change related to Ig sample. Values refer to biological replicates of three independent experiments. Bars represent mean values ± SEM. Significant differences and p-values were derived from an unpaired *t*-test, two-tailed. wt vs HEM or KO. **p-value<0.005.

References

81. Gazit R & Rossi DJ. Hematopoietic database: including HSCs, progenitors and effector cells. NCBIs Gene Expression Omnibus NCBIs Gene Expression Omnibus. 2015. http://www.ncbi.nlm.nih.gov/geo/.
