# Supplementary figures and images for: Single loss of a Trp53 allele triggers an increased oxidative, DNA damage and cytokine inflammatory responses through deregulation of IκBα expression
Source: Cell Death Dis. 2021 Apr 6;12(4):359. doi: 10.1038/s41419-021-03638-3 (PMC8024389; doi:10.1038/s41419-021-03638-3)

Figure S1.

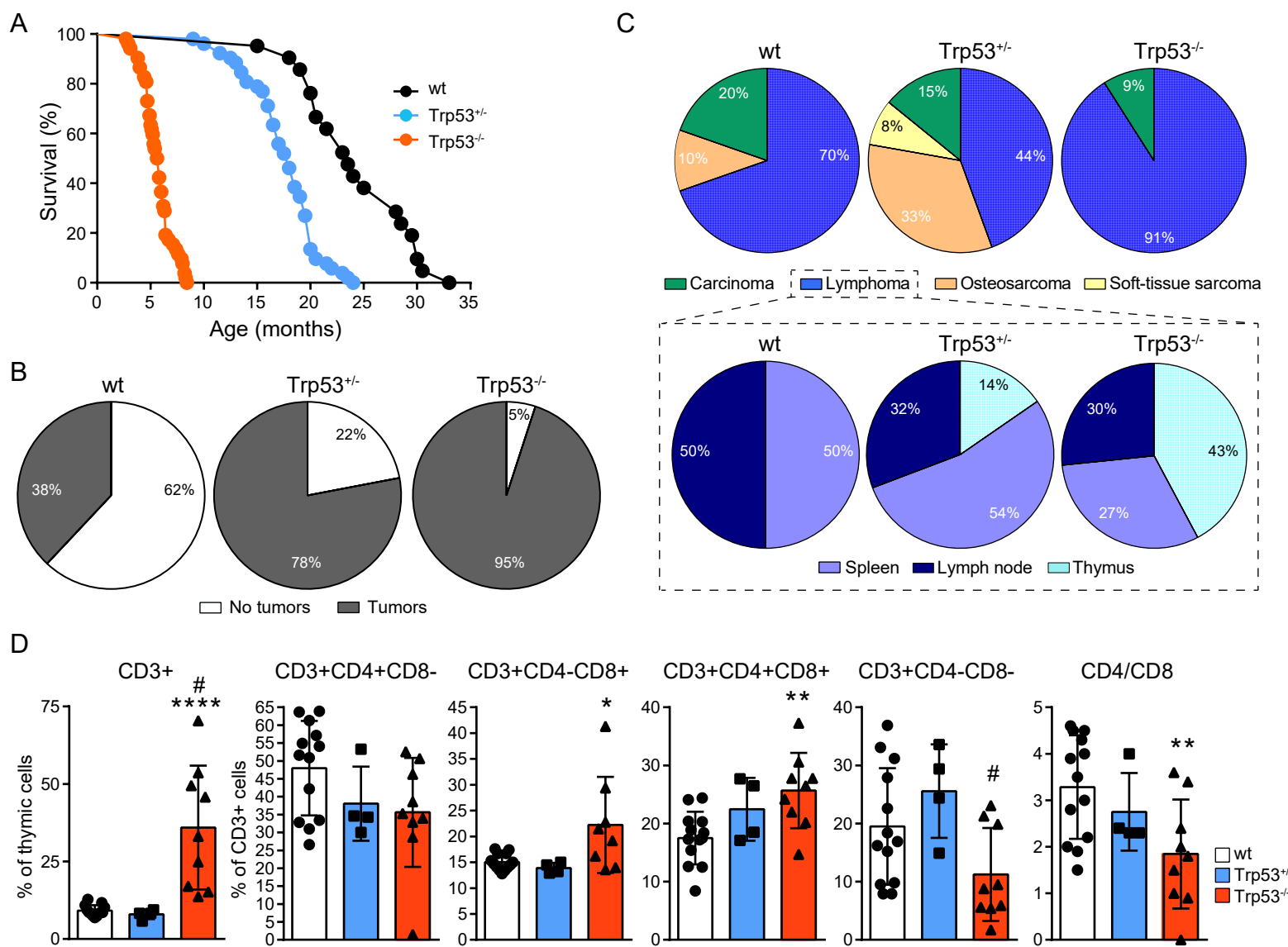

Supplement: Supplementary file 2 — Supplemental Figure S1 [file 41419_2021_3638_MOESM2_ESM.pdf]

Figure S2.

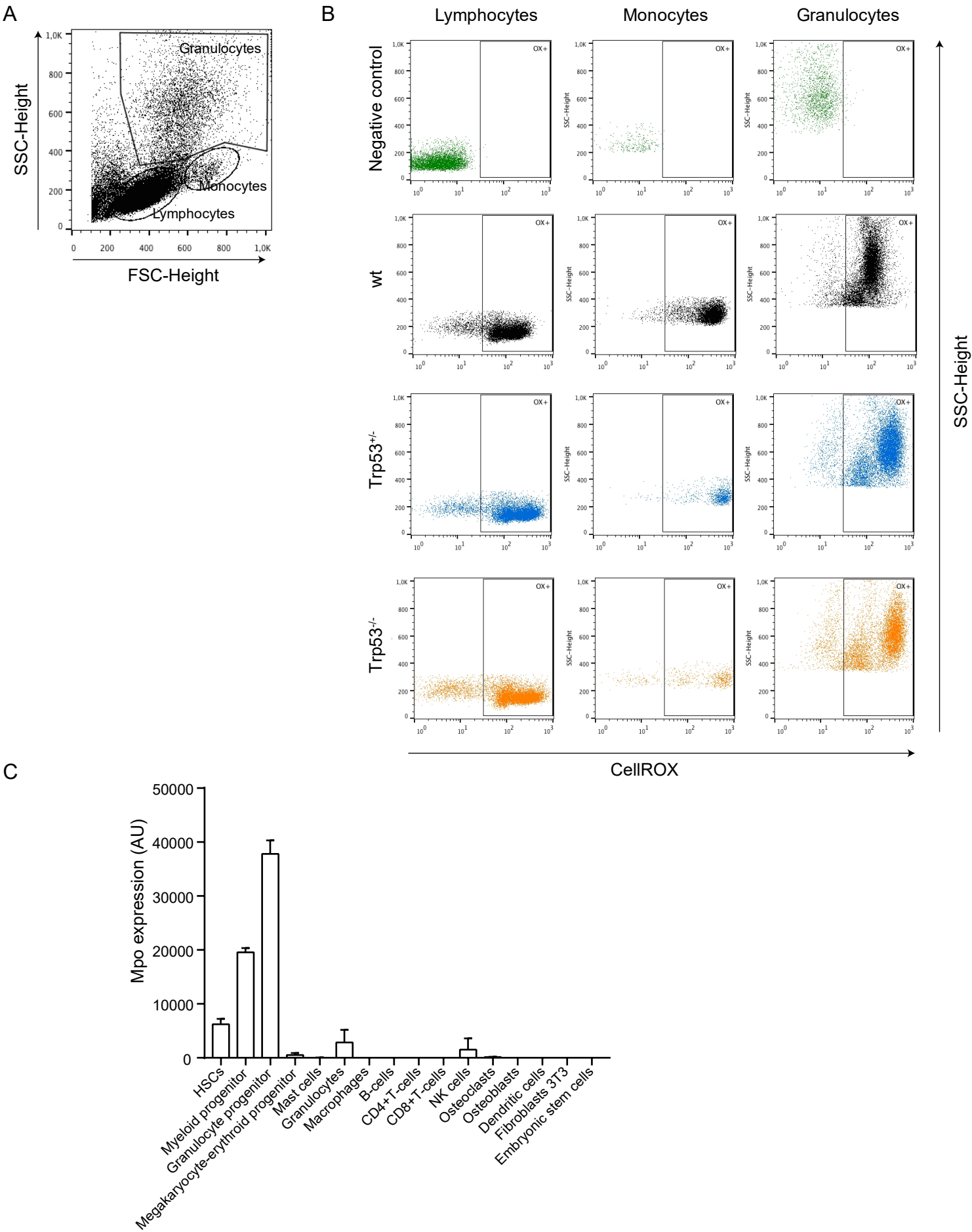

Supplement: Supplementary file 3 — Supplemental Figure S2 [file 41419_2021_3638_MOESM3_ESM.pdf]

Figure S3.

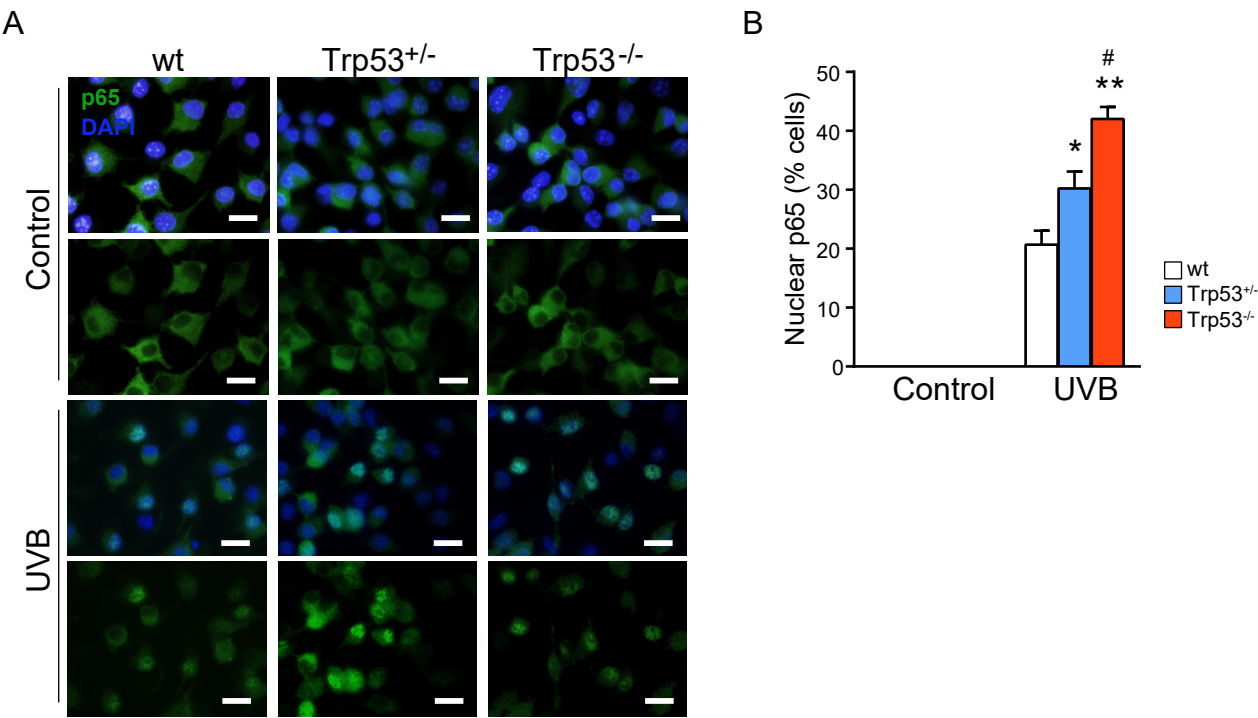

Supplement: Supplementary file 4 — Supplemental Figure S3 [file 41419_2021_3638_MOESM4_ESM.pdf]

Figure S4.

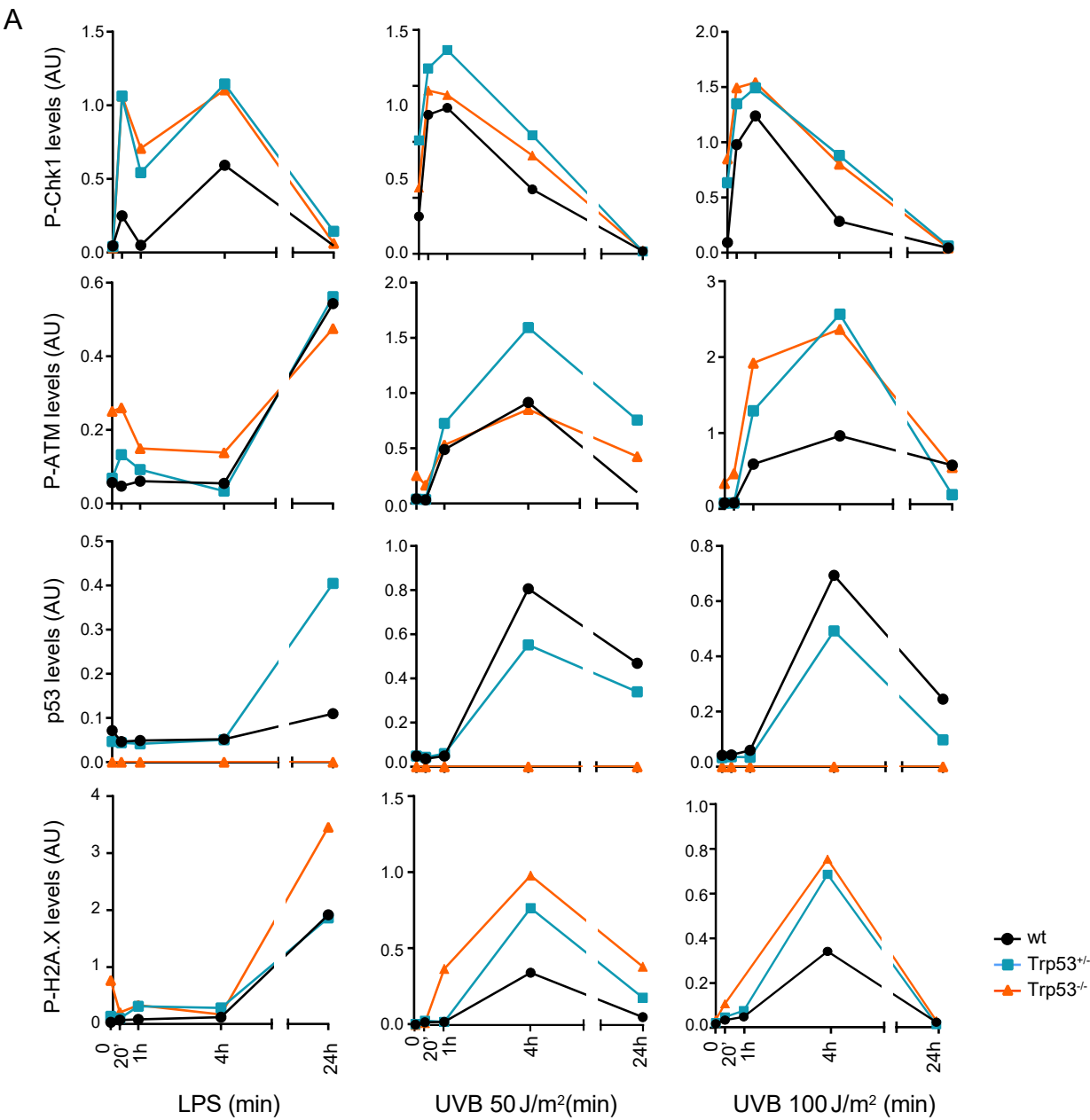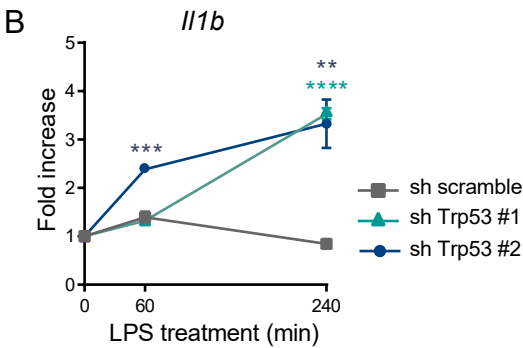

Supplement: Supplementary file 5 — Supplemental Figure S4 [file 41419_2021_3638_MOESM5_ESM.pdf]

Figure S5.

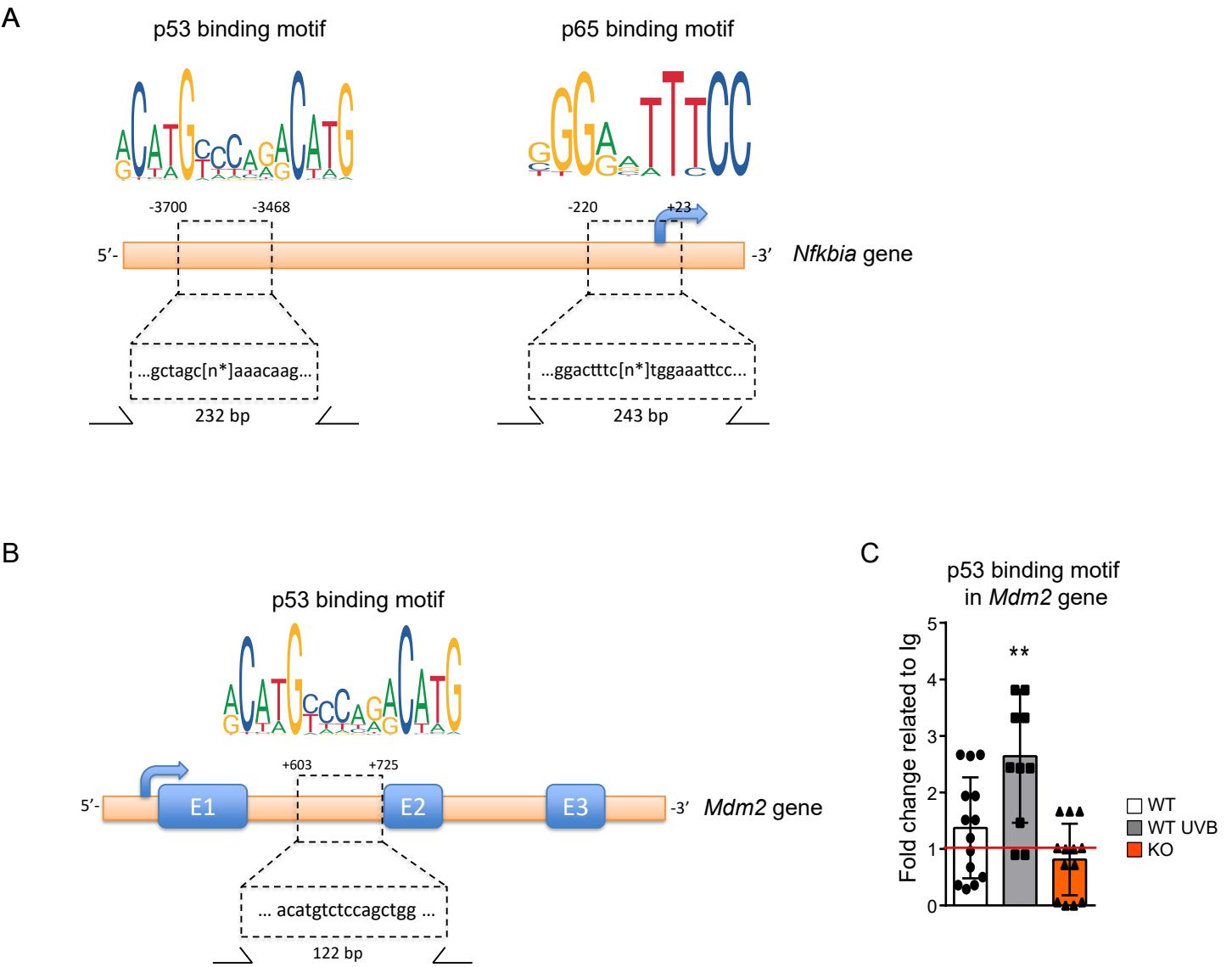

Supplement: Supplementary file 6 — Supplemental Figure S5 [file 41419_2021_3638_MOESM6_ESM.pdf]
